# Breastfeeding in infancy: identifying the program-relevant issues in Bangladesh

**DOI:** 10.1186/1746-4358-5-21

**Published:** 2010-11-30

**Authors:** Rukhsana Haider, Sabrina Rasheed, Tina G Sanghvi, Nazmul Hassan, Helena Pachon, Sanjeeda Islam, Chowdhury SB Jalal

**Affiliations:** 1 Training and Assistance for Health & Nutrition (TAHN) Foundation, Bangladesh; 2 Academy for Educational Development (AED), Bangladesh; 3 International Centre for Diarrhoeal Disease Research (ICDDR,B), Bangladesh; 4 Institute of Nutrition and Food Science, Dhaka University, Bangladesh; 5 Centro Internacional de Agricultura Tropical (CIAT), Colombia; 6 BRAC, Bangladesh

## Abstract

**Background:**

In Bangladesh, many programs and projects have been promoting breastfeeding since the late 1980 s. Breastfeeding practices, however, have not improved accordingly.

**Methods:**

For identifying program-relevant issues to improve breastfeeding in infancy, quantitative data were collected through visits to households (n = 356) in rural Chittagong and urban slums in Dhaka, and qualitative data from sub-samples by applying semi-structured in-depth interviews (n = 42), focus group discussions (n = 28), and opportunistic observations (n = 21). Trials of Improved Practices (TIPs) (n = 26) were conducted in the above sites and rural Sylhet to determine how best to design further interventions. Our analysis focused on five breastfeeding practices recommended by the World Health Organization: putting baby to the breast within the first hour of birth, feeding colostrum and not giving fluids, food or other substances in the first days of life, breastfeeding on demand, not feeding anything by bottle, and exclusive breastfeeding for the first six months.

**Results:**

The biggest gaps were found to be in putting baby to the breast within the first hour of birth (76% gap), feeding colostrum and not giving other fluids, foods or substances within the first three days (54% gap), and exclusive breastfeeding from birth through 180 days (90% gap). Lack of knowledge about dangers of delaying initiation beyond the first hour and giving other fluids, foods or substances, and the common perception of "insufficient milk" were main reasons given by mothers for these practices. Health workers had talked to only 8% of mothers about infant feeding during antenatal and immunization visits, and to 34% of mothers during sick child visits. The major providers of infant feeding information were grandmothers (28%).

**Conclusions:**

The findings showed that huge gaps continue to exist in breastfeeding behaviors, mostly due to lack of awareness as to why the recommended breastfeeding practices are beneficial, the risks of not practicing them, as well as how to practice them. Health workers' interactions for promoting and supporting optimal breastfeeding are extremely low. Counseling techniques should be used to reinforce specific, priority messages by health facility staff and community-based workers at all contact points with mothers of young infants.

## Background

While there is considerable evidence that exclusive breastfeeding contributes to child survival and that efforts to increase exclusive breastfeeding (EBF) have been effective [1-3], new evidence for the benefits of optimal early infant feeding practices continue to be documented. The impact of early initiation of breastfeeding on neonatal survival [4-7], and of EBF on cognitive development [8] provides further impetus to breastfeeding promotion programs. In Bangladesh, many programs and projects have been promoting breastfeeding for many years. From 1989 to 2002, the national recommendation was to breastfeed exclusively from birth to five months, but after endorsement of the Global Strategy for Infant and Young Child Feeding [9] by the World Health Organization's Member States in the World Health Assembly, the recommended duration of EBF was subsequently increased to six months.

Based on 24-hour recall reported in the national Bangladesh Demographic and Health surveys (BDHS) 1999, 2004, and 2007 [10-12], the prevalence of EBF (defined as giving only breast milk, not even water) among infants below six months of age has not increased in the past twelve years. The EBF rate remained unchanged at around 45% in the 1993 and 1999 surveys and then declined to 42% in the 2004 survey with no notable improvement thereafter. BDHS 2007 reports the mean duration of EBF to be 3.3 months. The median duration has remained the same in the three surveys: 1.8 months in 1999-2000, 1.7 months in 2004, and 1.8 months in 2007. Initiation of breastfeeding within one hour of birth, however, is reported to have increased from 24% in 2004 to 43% in 2007. Children born in a health facility or those whose births were attended by a health professional had a lower likelihood of breastfeeding within one hour of birth than those born at home. The tradition of giving pre-lacteal feeds (defined in the surveys as other liquids/foods given during the first three days of life) is widely practiced with 62% of newborns receiving such feeds. The most common early feeds consist of sugar or glucose water (42%), milk other than breast milk (36%), and honey (33%). The proportion of exclusively breastfed infants drops sharply from 52% at 2-3 months to 23% at 4-5 months. As with other DHS and MICS household surveys, the BDHS data is based on 24-hour recall, which tends to overstate the rate of EBF because it is not measured from birth.

Understanding the facilitators and barriers driving these patterns is an important first step towards preventing harmful feeding practices. Almost all Bangladeshi children are breastfed to some extent in the first year of life, and fortunately continuation of breastfeeding in the second year of life remains common, with 91% continuing to breastfeed [12]. And yet Bangladesh has one of the highest rates of malnutrition in South Asia with about half the children less than 5 years of age being underweight or stunted [12]. Addressing the issue of poor infant feeding practices in a holistic manner is an imperative. Two of the specific objectives of the National Strategy for Infant and Young Child Feeding [13], formulated in 2004 and to be achieved by 2010, are to increase the percentage of newborns who are breastfed within one hour of birth from 24% to 50%, and to increase the percentage of infants aged less than 6 months of age who are exclusively breastfed from 42% to 60%. Concerned that these objectives have not been achieved, and are unlikely to be achieved in the next five years at the current pace, we aimed to delve deeper into the reasons for mothers' infant feeding practices, and to use this information to identify and refine the training and communication strategies for effective community interventions and improved breastfeeding practices.

## Methods

The first step of the research was to undertake a situational assessment (March-April 2009), identify the gaps required for program planning, and then design the quantitative and qualitative aspects of the study to address these gaps as far as possible. This was followed by conducting a stakeholders' workshop in early May 2009. As we only needed to explore in-depth the gaps in the current program-relevant issues and the reasons for not practicing the recommended behaviors, it was suggested that a purposive/convenience sample could be chosen, and could use the recent national Bangladesh Demographic Health Survey (BDHS 2007) as the representative randomized survey for the quantitative data. So due to resource constraints, timing (coinciding with the rainy season), accessibility and need to move forward with an intervention design, we decided to use a purposive/convenience sample. A total of 360 (about 180 from each site) mothers with children below two years were calculated to be required for the quantitative general/household survey, with sufficient numbers in each age group (at least 30).

Approval for the formative research was obtained from the James P. Grant School of Public Health, Bangladesh Rural Advancement Committee (BRAC) University Ethical Review Committee. Written consent was obtained from all the study participants.

### Study sites

Stakeholders at the above mentioned workshop also suggested conducting the study in three areas, two rural, and one urban, with one of the rural areas in the country's northern region, and one in the southern region, based on high stunting rates (as per BDHS 2007), and in non-NNP (National Nutrition Program) areas. The urban area would be in Dhaka slums. Thus two locations - in rural Chittagong and in Dhaka slums - were chosen for the general/household survey. The specific sites were selected by the Field Managers in consultation with local BRAC officials and local community leaders. Considering accessibility and presence of many children under two years, the village *South Harina *in the sub-district *Lohagara*, Chittagong was selected as the first rural survey site. Later, a village in Sylhet (*Jaintapu*r) was selected for one of the TIPS sites. Similarly, local community leaders helped to identify particular sections of three slums in Dhaka (*Badda, Mohakahali Shaat tola and Mirpur*) for the urban location of the survey.

### Study participants

All participants were purposively selected for convenience and to assure they met certain criteria, which included geographical location in typical urban slums and rural low income communities; the minimum number of children needed for the analysis per age group of 0-2.9 months (n = 40), 3-5.9 months (n = 40), 6-11.9 months (n = 40), 12-17.9 months (n = 30), and 18-23.9 months (n = 30); a small, medium and large slum; and typical medium sized villages in rural areas. Other criteria were to include about 50% male and 50% female infants in the study, and both working and non-working mothers in the slums. The Field Managers identified and listed households with children below two years the day before so that the interviewers could start the survey from one end of the village/slum and continue till the quota for each age group of children and their mothers was completed.

For TIPs, only mothers with infants less than five months who were giving other fluids/foods in addition to breastfeeding were selected so that they could be counseled to stop these and switch to EBF. Their family members were encouraged to help with household chores so as to give the mothers more time for breastfeeding. (Other aspects related to complementary feeding in TIPS for older babies will be presented in a separate paper).

### Data collection

This cross-sectional study was conducted during June-August 2009. Women (majority with Masters Degrees) who had worked as interviewers for reputed organizations were recruited. They were divided into two teams, one for the quantitative, and one for the qualitative data collection, based on their experience. Each team consisted of six interviewers, along with one male field manager. Both teams were trained for two weeks on the different methodologies to be used (quantitative data collection or semi-structured and in-depth interviews, focus group discussions and opportunistic observations). They participated in the translation and field testing of all the data collection instruments. Although all had past experience in data collection, the techniques were refreshed over one week through role plays. During the second week the interviewers practiced using the quantitative questionnaires and qualitative formats in a slum in *Tongi *(outskirts of Dhaka city). The qualitative interviewers were paired into three groups based on their skills during field test so that if one person interviewed, the other person would observe and take notes. Most of the qualitative interviews were also recorded for verification.

Following *Pro*PAN [14], a series of ideal breastfeeding practices were identified and used to guide the analysis of quantitative and qualitative feeding data based on WHO documents [15-17]. For example, in the general survey, regarding breastfeeding on demand, mothers were asked, "do you breastfeed your child when he wants to or at fixed times?" If the mother answered "whenever the child wants to", the answer was classified as breastfeeding on demand.

The general/household survey questionnaire, semi-structured interviews, observations and TIPs data collection instruments were formulated based on the *Pro*PAN manual [14]. Accordingly, the EBF indicator was more strict as it calculates the proportion of children who never received anything but breast milk from birth to 180 days and the sample includes only children older than 180 days. The more widely used WHO indicator for this practice calculates 24 hour/current breastfeeding status of all infants from birth to 180 days [17]. The instruments were then modified to address the local context, translated into Bengali and field tested before finalizing. The quantitative general survey was administered to families of children from birth to 23 months old, and included questions on breastfeeding, complementary feeding, use of health services, access to health communications, housing, education, source of water and sanitation, and employment.

The qualitative data were collected from a sub-set of the same households which participated in the quantitative survey. This was done through semi-structured interviews with mothers, caregivers, traditional birth attendants, village doctors, and group discussions with grandmothers and fathers, all focused on reasons why mothers and caregivers practice certain breastfeeding behaviors. Opportunistic observations were carried out during qualitative and quantitative interviews to observe breastfeeding techniques, specifically position and attachment of the baby at the breast, and type of feeding utensils used if additional fluids or foods were given. After a rapid analysis of both qualitative and quantitative information, some specific messages were selected. These messages were tried out through TIPs. Families of infants aged to five months, selected in an opportunistic manner, were asked to try to modify one or two feeding practices (recommendations) appropriate for their child's age (such as not giving water and practicing exclusive breastfeeding, and family members helping with household chores) during the first visit. In the second visit three to four days later, the same households were visited to assess their experience of trying out the given recommendation. For each practice that was tested, the responses of families were organized in a matrix that showed whether or not the respondent remembered the recommended practice, had tried it, liked it, and would continue it. The reason for each response was also recorded. Motivating factors and barriers as reported by families were listed. During analysis, reasons for accepting or not accepting each tested recommendation were ranked by how many families gave the same reason.

### Data analysis

Quantitative data were entered and analyzed using *Pro*PAN's Epi Info-based statistical program (Version 6) for the descriptive statistics (shown in Table 1). The qualitative data was analyzed manually, based on the recommended feeding practices defined by WHO, using the *Pro*PAN methodology. Behaviors and practices mean the same in this paper, as it does in the *Pro*PAN module. The themes were then triangulated (such as for initiation of breastfeeding, feeding of other liquids/foods during first three days, and exclusive breastfeeding) using results obtained by different methods and from different sources of information.

## Results

Mothers with infants aged from birth to 23.9 months from 356 households participated in the quantitative household survey. There were 42 semi-structured interviews and 21 opportunistic observations recorded for this age group. Fifteen focus group discussions were held with grandmothers and 14 with fathers. TIPs were conducted with mothers of 26 children aged from birth to 5.9 months.

### Quantitative information

#### *Socio-demographic information*

Among the 356 households surveyed, 176 (49%) were in the rural area and 180 (51%) in the urban slums. The mean age of the mothers was 25 years and mean age of the children was 9 months (Table 1). Among the mothers, 253/356 (71%) said they had given birth to their last child at home. About 75% of these mothers had houses with tin roofs, 53% cement floors, 45% mud floors, 96% toilets, 81% electricity, 68% mobile phones and 11% radios. Although only 43% had a TV, a large proportion watched TV in community groups. Among the mothers of children under two years, 98% were married, and 76% said they could read and write. Sixty-five percent of the fathers were hired workers.

**Table 1:** Socio-demographic information of households in the general survey (N = 356)

Characteristics	mean (SD) or n (%)
Mother's age (years)	24.98 (5.3)
Mother's education, completed school years	5.54 (3.9)
Antenatal visits	2.94 (2.5)
Baby delivered	
at home	257(72.2)
in health facility	99 (27.8)
Male infants	167 (46.9)
Female infants	189 (53.1)
Child's age (months)	8.66 (6.8)
Persons in the household	5.51 (2.5)
Children below 5 years of age	1.42 (0.6)
Father's education, completed school years	7.22 (10.5)
House roof, tin	268 (75.3)
House floor, cement	192 (53.9)
Electricity in the home	287 (80.6)
Number of possessions^1^	
1-4	186 (52.2)
5-8	169 (47.5)
9-12	0 (0)

^1 ^Out of 0-12 score based on reported possession of the following: mobile telephone, fixed telephone, almirah or wardrobe, table, chair, watch, bicycle, motorcycle, motor scooter or tempo, animal-drawn cart, car or truck, boat with a motor, or rickshaw/van.

Our results focus on five breastfeeding practices recommended by WHO [14]. These were: putting baby to the breast within the first hour of birth; feeding colostrum and not giving other fluids, food or other substances in the first three days of life; breastfeeding on demand; not giving any type of milk, thin gruel, water, juices or sugary drinks by bottle and teat in addition to breastfeeds; and exclusive breastfeeding for six months. WHO also recommends continued breastfeeding for two years, but we did not elaborate on this practice as it is common in Bangladesh.

#### *Gaps in practicing recommended behaviors*

As seen in Table 2, the biggest gaps in recommended breastfeeding practices were in putting baby to the breast within the first hour of birth (76% gap); giving colostrum and not giving other fluids/foods in the first three days (54% gap); and exclusive breastfeeding from birth through 180 days (90% gap). Breastfeeding on demand (96%) and not bottle feeding (85%) did not have big gaps and so were not of concern.

**Table 2:** Prevalence of mothers practicing recommended behaviors (N = 356)

Recommended behaviors	n (%)
Breastfeeding initiation within first hour after birth	87 (24)
Giving colostrum, and not giving other fluids or foods in the first 3 days of life	163 (46)
Not bottle feeding	301 (85)
Breastfeeding on demand	324 (96)
Exclusive breastfeeding from birth through 6 months*	20/197 (10)

*This indicator is more strict as it calculates % of children who never received anything but breast milk from birth to 180 days and the sample includes only children >180 days. The more widely used indicator for this practice calculates 24 hour/current breastfeeding status of all infants 0-180 days.

#### *Information sources for infant feeding*

About 295/356 (83%) of mothers reported receiving information on breastfeeding in their own home or community, and only 57/356 (16%) in a health facility. Table 3 presents information on sources and types of information on infant feeding recalled by mothers. Family members (36%) were among the persons who commonly talked to mothers about infant feeding, while health personnel (18%) played a lesser role. Mothers reported that they obtained information regarding infant feeding mostly from the media (81%), and of those who recalled media messages, television (97%) was the main source. The most commonly recalled messages (from TV and other sources) about breastfeeding and complementary feeding included "give only breast milk for six months" (31%); "feed the child other foods after six months of age" (22%)" and "feed the child *khichuri *after six months" [*khichuri *is a soft mixture of rice, lentils, vegetables, cooked with little oil and spices to which egg, meat or fish may be added] (16%). Although 31% of mothers had heard about exclusive breastfeeding for six months, very few (7%) could recall being told that "colostrum should be fed right after birth".

**Table 3:** Common sources and types of information on infant feeding in the study area (N = 356)

Sources and information heard	n (%)
Mothers who had received infant feeding information	295/356 (83%)
	
Person who had talked most about infant feeding	
Family member	107/295 (36)
Neighbor	99/295 (33)
Health personnel	55/295 (18)
	
Considered most reliable source of information on infant feeding	
Grandmother	103/356 (28)
Doctor	87/356 (24)
Health personnel	62/356 (17)
	
Remembered hearing message about infant feeding from mass media (radio, TV, newspaper, magazine)	285/356 (81)
Of those who heard, from radio	30/285 (11)
Of those who heard, from TV	278/285 (97)
	
Messages about practices recalled from mass media**	
Give only breast milk until 6 months	84/272(31)
Give other food after 6 months along with breast milk	60/272 (22)
Feed the child *khichuri≠*	43/272(16)
Feed colostrum right after birth	18/272 (7)

**Commercial advertisements about specific products also heard/seen

± *khichuri - *soft mixture of rice, lentils, vegetables, cooked with little oil and spices to which egg, meat or fish may be added

#### *Health workers' role*

Table 4 shows health workers' contributions in guiding mothers about infant feeding. About 21% of the mothers had more than four antenatal checks, but few mothers (8%) recalled the providers mentioning anything about breastfeeding or complementary feeding. Similarly, a large proportion of children below two years were seen by health workers at multiple contacts for immunizations (nationwide 88% complete three doses of DPT) [12] but again, few mothers (8%) reported receiving any information about breastfeeding or complementary feeding. Mothers (34%) recalled information given during sick child visits, perhaps because the country's Integrated Management of Childhood Illness (IMCI) program includes training for health providers on infant feeding practices.

**Table 4:** Health workers' role in informing mothers about infant feeding (N = 272)

Contact with health workers	n (%)
Health worker or anyone from health center talked to you about breastfeeding or CF during:	
antenatal care	24 (8)
during immunization	25 (8)
during sick child visits	104 (34)
	
Specific messages remembered being given by health workers:	
"Continue breastfeeding even if baby sick"	28 (22)
"Have to feed breast milk"	14 (11)
"Should not give any other food before 6 months	15 (12)
"Should give variety of foods (to improve appetite)"	14 (11)

BF = breastfeeding, CF = complementary feeding

### Qualitative information

To explore why gaps exist in the three critical areas highlighted above (putting baby to the breast within the first hour of birth; not giving other fluids/foods within the first three days and giving colostrum; exclusive breastfeeding from birth through 180 days) and how they can be filled, mothers' experiences with access to information on breastfeeding and reasons for practising or not practising the recommended behaviors were discussed in detail. This qualitative information on barriers and motivations that influence the key behaviors is obtained from semi-structured interviews and focus group discussions and summarized in Table 5 and 6 and in the text below (along with supportive quantitative data).

**Table 5:** Barriers and facilitators* for giving breast milk within one hour of birth and for not giving other fluids or foods within the first three days of life

	Barriers		Facilitators	
**Recommended** **Practice**	**Internal**	**External**	**Internal**	**External**

**Giving breast milk within one hour of birth**	- did not know that baby has to be given breast milk first - put baby to breast but there was no milk - tried to breastfeed 6 hours later - milk came in late - placenta delivered late	- mother and child have to be bathed first/relatives take time - mother not well/unconscious/doctor took time to examine the mother - delay in bringing baby to be fed/caesarean, twin births - grandmother's decision about what to give when/no milk - baby was ill/weak, unable to suck - no one helped - midwife discouraged breastfeeding for first 3 days	- knew that it is important to breast- feed as soon as possible - told by health center staff/doctor about giving breast milk immediately	- mother was well after delivery so could start breastfeeding - doctor said to immediately put baby to breast - normal delivery - had caesarean but nurses brought baby to feed immediately

**Not giving other fluids or foods within the first** **three days of life**	- did not know that other liquids should not be given - knows that milk does not come for 3 days/then something must be given - heard that substances like sugar water (to prevent throat from drying), honey (for sweet words/preventing illness), mustard oil (to clean mouth) are required to be given	- older women know better- advise honey - mother was ill, so others gave honey/doctor gave oral saline to baby - everyone gives other fluids first - TBA^± ^advised sugar water as baby's throat dries - grandmother cleaned mouth with mustard oil to clear impurities, then gave honey - honey/glucose water given as milk did not come - village doctor gave glucose water - mothers do not decide what to give	- mother knew about giving breast milk first from TV, doctors and health workers	- doctor did not allow/said ok not to give anything for few hours - milk came so other fluid was not given - grandmother said not to give anything/causes disease/was bad - there was nothing else available to give the baby

*Internal barriers/facilitators are those factors relating to knowledge, attitude, skills and psychological state which are in the mother's control. External barriers/facilitators are factors over which mothers don't have any control [14]. ^±^TBA = traditional birth attendant (maybe trained or untrained in safe delivery methods)

**Table 6:** Barriers and facilitators for six months of exclusive breastfeeding (EBF)

Barriers		Facilitators	
**Internal**	**External**	**Internal**	**External**

- thinks that without water baby will be thirsty/will not be able to digest anything/won't live/spirit will die - did not know water feeding is harmful - learnt about EBF later - fruit juice is good for health - should be fed everything from 3 months or later baby will not eat other foods	- baby wanted to drink water - baby did not get enough breast milk baby cried/did not want to breastfeed - mother-in-law and/husband advised water/fruit juice - doctor said to give water with breast milk/prescribed formula for twins - contraceptives/antibiotics/inadequate consumption of good food reduced milk	- feeding water may cause baby to catch cold	- had adequate milk - baby had mouth sores so could not have anything else but breast milk

### *Recommended practice: putting infant to breast within one hour of birth*

Only a quarter of the mothers reported initiating breastfeeding within one hour of birth, while the majority did so within one to three hours. Timing of initiation did not differ if the mother could read and write, was involved in income-generating activities, or was employed outside the home. After probing, most mothers who had vaginal deliveries could generally remember when in the process of tying the cord, cleaning up and delivering the placenta that the infant was placed on her breast and started suckling. In the case of caesarean deliveries or other complications, however, mothers did not know when breastfeeding was initiated, and either the grandmother, aunt of the baby or neighbor who had been present at the time of birth, had to be asked. It was noted that often the mother and/or the family members were not aware that breastfeeding should start immediately, or they thought there was no milk at that time (Table 5). The general perception was that milk does not "come down" for one to three days so when the baby cried after being breastfed, it reinforced this perception and other liquids/foods were fed. In addition, as drops of white milk were not seen around the baby's mouth during or after breastfeeding, this further reinforced the false beliefs that colostrum is not sufficient.

### *Recommended practice: feeding colostrum and not giving other fluids or foods within first three days*

In-depth interviews showed that the only barriers for feeding colostrum were that "it looked bad" or "grandmother advised not to give". Most mothers said that it was good to give colostrum as "it protects baby from disease and makes the child smart", which they had heard from mother-in-law, TV, doctor, traditional birth attendant (TBA), family planning worker, garment factory worker, BRAC and health center workers. Nurses had said it was "like the first immunization (*teekka*)". Some mothers talked about feeding colostrum as the norm, having heard from others in their social network that "everyone feeds colostrum now". Other replies included "older sister had fed colostrum" and "grandmother helped to feed". One mother said "baby did not cry after feeding colostrum", implying satisfaction of the baby's need. Lack of knowledge that other fluids are unnecessary and harmful, the misperception that breast milk does not come down in the first three days or is insufficient for the baby's needs, or that water is essential were the common reasons for providing the other fluids/foods as shown in Table 5.

The barriers which delayed early initiation of breastfeeding (Table 5) can thus be summarized as follows:

*Maternal barriers *- Mother had limited knowledge and awareness of the importance of early initiation of breastfeeding; mother did not decide to initiate breastfeeding because of difficult or prolonged labor and unconscious state;

*Infant barriers *- the inability of baby to suck, or baby's illness after birth;

*Barriers related to support *- bathing baby and mother just after birth, thus delaying breastfeeding initiation, decision by grandmothers or other family members to give other fluids and foods in the early days, and discouragement for early initiation of breastfeeding by traditional birth attendants.

### *Recommended practice: six months of exclusive breastfeeding*

There were a number of reasons why EBF could not be practiced by mothers for the first six months (Table 6). Many of these, as mentioned by mothers and family members, were related to a belief in the need for water: "without water baby will be thirsty", "baby won't live without water", and "without water the spirit dies". Others said they did not know that feeding water was harmful. Family members (fathers and grandmothers) were noted as saying "doctor said to give water and breast milk", or "also give other milk along with breast milk". While "baby did not get adequate milk" was the most frequent reason given by mothers, "baby crying" was taken as an indicator for insufficient milk, or "baby not wanting to breastfeed". Again, it was a common belief that the supply of breast milk was insufficient, and knowledge that milk supply could be increased by more frequent suckling was lacking. As one grandmother said, "She (daughter-in-law) did not have milk, and baby cried a lot. We took her to the doctor and he advisede '*potter dudh*' (tinned milk). If there is no milk, what can be done?"

Further probing during TIPs to find out why mothers thought that they had "insufficient milk" brought a response like this: "*Neejaye toe bujhi - buk khali khali lagay, dudh bhalo bhabay ashlay, buk bhora bhora lagay*" (I can understand - breast feels empty, if milk flows well, breast feels full). This is a common example of a false belief. Mothers often worry that they have insufficient milk because their breasts feel "empty", however it is normal after a few weeks of breastfeeding for the breasts to cease feeling full before a feed; this does not mean that the breasts are "empty". On the contrary, mothers can be reassured that after few days the milk supply is usually well established and large amounts of breast milk can be produced by the breasts. Other responses were: "*Baccha baar baar khaitay chai*"(baby wants to feed frequently); "*Baccha kaandey*"(baby cries); "*Paara protibeshi bolay baccha kay alga dudh dao - baccha ghumabay aar tumi shanty tay thakba - aar onno kaaj kortay parba*"(neighbors say to feed baby other milk - he will sleep, and you will be at peace and have time to do other work).

Some mothers reported "inadequate consumption of good food reduced milk" - good food meaning meat (beef or mutton), chicken, big fish and so on which again is a false belief because it's the amount of food/caloric intake which affects breast milk production, not the type of food she eats. A few mothers reported that "antibiotics taken during illness decreased milk supply" which is also unlikely. However, the belief expressed by some mothers regarding "use of contraceptives reduced milk supply" cannot be discarded as the oral combined estrogen-progestin containing pill is commonly given to postpartum mothers and reported to reduce milk output. Some mothers reported that "returning to work before the baby reached six months prevented me from breastfeeding my baby exclusively." Other mothers said they heard the message about EBF after they had already started other foods.

The only responses were given in support for EBF: "mother had plenty of breast milk" or "baby had mouth sores so could not eat anything else".

Interactions of health workers with urban working mothers of sick children have given the general impression that they do not try to breastfeed (personal communication). The responses we obtained from the urban working mothers in our study area, however, indicated that they found it difficult to breastfeed exclusively because they were away from their homes for long periods during the day. Some strategies these mothers used included: bringing adult family members from the village to feed and care for the baby during mother's absence, and breastfeeding when she returned home; employing people to care for the baby and to bring the baby to the workplace to be breastfed, or returning home to breastfeed during the work day; or taking the baby to work along with an older sibling who looked after the baby (case of a day laborer); or leaving employment at a factory for home-based work (making paper bags) which allowed the mother to engage in childcare and breastfeeding.

Opportunistic observations of infants being breastfed showed poor position and attachment, mothers rocking the baby while trying to breastfeed, switching breasts during one feed without waiting for baby to finish feeding from one breast, and very short duration of breastfeeds removing baby from the breast while he was still suckling. Although short duration of feeding will supply the foremilk to quench the baby's thirst, interruption of a breastfeed will prevent the baby from getting the richer hind-milk to satisfy his caloric needs.

As it was not possible to influence initiation of breastfeeding during this research (due to insufficient time to follow-up pregnant women), TIPs could only focus on encouraging mothers to convert to EBF if they were predominantly or partially breastfeeding, and for family members to take a bigger share of the household chores. Twenty six mothers of infants below five months of age agreed to try out the recommended breastfeeding practices, 25 mothers remembered and tried them, and all 25 mothers liked the change and intended to continue. The statements made by the mothers that made EBF easier were that "feeding only breast milk to younger babies will be good for their health" (n = 14), "I have sufficient milk" (n = 11), "neighbors/family are encouraging" (n = 7), and "baby is responding well" (n = 4). Mothers also mentioned that "breastfeeding saves money" (n = 3), "saves time" (n = 2), "develops the brain" (n = 1), and is "easy to digest" (n = 1). These mothers said that after the benefits of EBF were explained and they were shown "how" to have sufficient breast milk, they felt confident about sustaining the changed practice until the baby was six months old.

The few difficulties faced by mothers for EBF identified during TIPs were mother's illness (n = 3), mother had to send baby to grandparent's house (n = 1), caesarean-section delivery (n = 2), premature baby (n = 2), and as baby was sick, felt breast milk may not be adequate so gave *suji *[gruel made with rice or wheat powder, with or without milk and sugar] (n = 1).

## Discussion

This study extends further knowledge in the field of breastfeeding in infancy by documenting mothers' perceptions that delay timely breastfeeding initiation and interfere with EBF. Although many mothers had heard some breastfeeding promotional messages from the media and other sources, these did not sufficiently influence their perceptions and practices. This resulted in big gaps in initiating breastfeeding within one hour, feeding colostrum and not giving other fluids/foods within the first three days, and in exclusive breastfeeding for six months. The abysmally low level of interaction by health workers despite multiple opportunities provided during antenatal, immunization and sick child visits, and the absence of well informed skilled support at community level, were contributory factors.

Among the recommended breastfeeding practices however, the prevalence of colostrum feeding is reported to have increased over the years, from 87% to 92% [11,12], which may partly be attributable to the promotional message that emphasized its importance by calling it the "first vaccination" and also by the increase in initiation of breastfeeding within one hour (from 24% to 42%) [11,12]. It may also be that since colostrum is often termed the "first milk" or "*prothom dudh*", only the first few drops of colostrum are fed as intake of other fluids and foods during the first three days is still high, 62% nationally [12], and 54% in our study. Thus it is obvious that there is a large knowledge-to-practice gap and that the media messages have not been clearly focused on the specific recommendations and how they can be implemented. The same applied to the health workers, who either did not prioritize the relevant infant feeding messages, or missed opportunities with mothers to promote and support optimal breastfeeding practices. The low prevalence of information on breastfeeding given by health workers at antenatal visits (and other visits - immunization, sick child visits) is probably due to the fact that it is generally not included in the practical training of reproductive and other health workers, both in the government health system and non-government programs although it might be there in the theoretical training. There is thus an urgent need for updating the in-service and pre-service curricula, as well as the training manuals, with particular emphasis on practical sessions. Standardized accreditation requirements are lacking, and health staff is not required to take refresher exams. In addition, there is no indicator for assessing and monitoring performance of health workers regarding infant and young child feeding.

Appropriate counseling and negotiation techniques used by interviewers' meeting mothers for the first time during the TIPs could convince them to convert to EBF in a few days. Behavior change was achieved when benefits that were meaningful for mothers were emphasized. The most frequently mentioned benefits in order of priority, were benefits for child's physical and brain development, protection from illnesses, baby's safety, and mother's convenience. Other benefits included ability to learn simple strategies to prevent and overcome common difficulties (such as through correct positioning and attachment and manual expression of breast milk), and the ability to accurately self-assess adequacy of breast milk supply and understand how to maintain adequate supply.

The strength of this formative research was that quantitative and qualitative data were collected simultaneously for understanding infant feeding practices along with the barriers and facilitators for following recommended practices. We did not dwell on what was already known in the country, but tried to add to this knowledge by exploring in depth the gaps in existing information. One of the limitations was that the sample may not have been representative of the whole population, although our results seem quite similar to the national BDHS 2007 data. Another limitation was that since the *Pro*PAN methodology was used, 24 hour recall for infants below six months was not collected. If problems occurred during that six month time frame, relevant information may not have been collected and captured through the qualitative methods used.

Caregivers' perceptions related to breastfeeding have been documented by studies [18-20]. It was reported earlier that breastfeeding messages were not clear [21], and there seems to be minimal changes in perceptions and practices between 1995 and 2009, except for the awareness about benefits of colostrum, and its reported intake. The question then is, why are there are still so many barriers to early initiation of breastfeeding if colostrum feeding prevalence has increased? Why are fluids that were traditionally fed or put in the mouth in the first three days before starting breastfeeding, now given after colostrum has been fed? And what are the socio-cultural reasons for why EBF prevalence is not improving? These are urgent issues for further in-depth research.

Considering the barriers for early initiation of breastfeeding (Table 5), it was obvious that the recommendation regarding colostrum feeding is more acceptable than that of not giving any other fluids except breast milk after birth. Mothers seem to be ill informed about the critical importance of timely suckling immediately after birth, not only to ensure that the baby benefits from colostrum, but also to facilitate milk let down, provide warmth through contact with the mother's body, promote uterine contractions, reduce blood loss, help delivery of the placenta and ensure survival of the newborn. Thus knowledge and awareness about this issue is extremely important before the baby is born, as well as the support of people around the mother to facilitate early initiation. Antenatal visits and interactions with birth attendants are important missed opportunities for encouraging optimal breastfeeding practices.

Since a stricter criteria than the WHO 24-hour recall indicators was used to assess the prevalence of EBF for the total duration of the first six months, the figure reported here (10%) is much lower than those reported in national surveys, but similar to low rates reported from other studies [22,23]. One study showed that better household food security status was associated with poor infant feeding practices during 3 to 6 months, but with better practices during 6 to 12 months [23]. We wonder if EBF is considered only a "theoretical or idealized" recommendation, and why strategies that have worked in Bangladesh, such as peer counselors for increasing EBF [24], or postnatal visits by trained community health workers to improve breastfeeding initiation of newborns [25] have not been utilized. While recognizing the significance of exclusive breastfeeding for child survival [26-29], and evidence for effective affordable strategies as mentioned above, it is pertinent that public health personnel ask why these strategies have not been more widely implemented in Bangladesh (and elsewhere). EBF promotion through lay counselors and community health workers has received very little investment and attention globally and has not been a priority for the major funders (compared to interventions such as vaccination, Vitamin A supplementation etc). While promotion and support of EBF requires appropriate training of lay counselors and community health workers to provide them the skills for effective interpersonal counseling, it also requires supportive supervision by similarly skilled and knowledgeable supervisors. Above all, it requires the managers of health programs and the donors to believe that EBF is truly possible. It must be appreciated that achieving and maintaining exclusive breastfeeding requires behavior change not only by the mothers but also by her family members and subsequently the community. Breastfeeding counseling is not a one-time event like giving a vaccine shot or a Vitamin A capsule, nor is it a marketable commodity. Other detriments are the absence of hands-on practical sessions during training, unrealistic targets of households to cover by each worker, which are not feasible, and not keeping an eye on results and feeding it back into the program. Mothers (and health workers) need frequent reinforcement and encouragement to sustain the changed behaviors which is often overlooked in many programs. Although lay counselors paid a small honorarium, or volunteers given some incentives can do this for mothers, the sustainability of such projects or programs is often questioned. It is still not clear, however, that if child health and survival is a goal to be achieved, why most donors hesitate to pay for these basic and essential services for ensuring appropriate nutrition of infants and young children, without which this goal cannot be achieved.

It is only recently that some non-government organizations (NGOs) in Bangladesh (BRAC, Save the Children USA) are trying to take their health and nutrition projects to scale using the concept of lay counselors (as infant and young child feeding promoters) and training selected community health workers to provide sustained support for infant feeding.

### Implications for policies and programs

Our findings have several implications for public health policy and program implementation. Specific and priority messages need to be selected and reinforced by health providers who are a major influence, both in rural and urban areas. Village doctors in particular should be deterred from advising early complementary feeding to address "insufficient milk". Training of health workers (facility and community-based) and volunteers should focus on counseling rather than on just giving messages. The focus should also be on "how to" breastfeed, rather than telling mothers "what" to do. The following must be clearly communicated and emphasized: dangers of not initiating breastfeeding within one hour for child survival and health, and dangers of giving other fluids and foods within the first three days (and in the first 6 months). Other topics that need to be communicated clearly during the training (and practiced) are the importance of proper positioning and attachment of the baby at the breast, why and how to express breast milk, and when to refer to a trained skilled provider if the mother has doubts or problems. Myths and misconceptions should be addressed in a culturally sensitive manner, utilizing various modes and channels of communication. As visual communication messages are remembered, more efforts must be made to show these educational messages in videos or mobile vans which could be screened in community gatherings, schools, clubs, etc. The timing for showing such educational messages in TV spots is also important - should preferably be just before, or in the middle of popular TV shows or films which are watched by mothers.

Essential breastfeeding education must start early in the antenatal checks, as early as the first or second trimester, in case the pregnant woman does not return to the health facility or to a group education session in the community. Other opportunities for contacts of health workers with mothers such as immunization, family planning, and sick child visits need to be used to encourage optimal breastfeeding practices. EBF should be strongly encouraged and can be easily adopted if supported. Motivators for mothers such as "breast milk is good for child's physical and brain development" and "breast milk protects from illness" should be emphasized, while informing them that most mothers can produce sufficient milk for their babies. At the same time, difficulties and problems must be given attention, such as "perception of insufficient milk" and "inability to breastfeed exclusively if mother or child is ill". Grandmothers, both maternal and paternal, must be targeted to obtain their support, as has been identified from studies in other countries [30]. Reinforcement and encouragement by doctors and family members are key to successful breastfeeding. In urban areas, neighbors can also play a supportive role. Women's work is not the main deterrent for desirable breastfeeding practices at this time but as the proportion of mothers who work outside the home rises, attention must also be paid to establish breastfeeding-friendly work sites.

Suboptimal breastfeeding practices have significant mortality consequences worldwide. The 2003 Lancet series stated that among the preventable child death interventions, breastfeeding alone could prevent 13% of deaths, the highest figure in the list [26]. In another report, the potential number of lives saved through improved breastfeeding practices is 1.4 million [27], similar to a previous estimate using a different categorization of breastfeeding practices (combining predominant and partial breastfeeding) and different mortality risks [28,29]. The World Health Organization's Member Countries, along with national and international partners, have reaffirmed their commitment to further reduce infant and child mortality through achieving the Millennium Development Goals by 2015. It is unlikely, however, that the goal to reduce by two-thirds the mortality of children under five will be met, unless neonatal mortality is halved. At the core of these interventions for survival and healthy development during the first weeks of life is early and exclusive breastfeeding, achievable even in resource poor settings. One in every five deaths can be prevented through timely initiation alone [4-6] and by EBF thereafter [31]. Ensuring early and exclusive breastfeeding is thus crucial for the survival of newborns and young infants, and for their healthy development.

## Conclusions

The research findings showed that huge knowledge-to-practice gaps continue to exist in breastfeeding behaviors, mostly due to lack of awareness as to why the recommended breastfeeding practices are beneficial, the risks of not practicing them, as well as how to practice them. Health workers' interactions for promoting and supporting optimal breastfeeding are extremely low. These findings have implications for public health policy and program implementation. Counseling techniques should be used to reinforce specific, priority messages by health facility staff and community-based workers at all contact points with mothers of young infants.

## Competing interests

All the authors declare that they have no competing interests. RH was former Regional Adviser, Nutrition, at the WHO South-East Asia Regional office in Delhi.

## Authors' contributions

All the authors have made substantial contributions to conception, design and acquisition of data, and in the analysis and interpretation of data; RH drafted the manuscript, TS, SR, HP were involved in revising it critically for important intellectual content; and all authors gave final approval of the version to be published.
